# Human severe sepsis cytokine mixture increases β2-integrin-dependent polymorphonuclear leukocyte adhesion to cerebral microvascular endothelial cells *in vitro*

**DOI:** 10.1186/s13054-015-0883-z

**Published:** 2015-04-07

**Authors:** Chris Blom, Brittany L Deller, Douglas D Fraser, Eric K Patterson, Claudio M Martin, Bryan Young, Patricia C Liaw, Payam Yazdan-Ashoori, Angelica Ortiz, Brian Webb, Greg Kilmer, David E Carter, Gediminas Cepinskas

**Affiliations:** Department of Physiology and Pharmacology, Western University, 1151 Richmond Str. North, London, ON N6A 5C1 Canada; Children’s Health Research Institute, 800 Commissioners Road East, London, ON N6C 2V5 Canada; Centre for Critical Illness Research, Lawson Health Research Institute, 800 Commissioners Rd East, London, ON N6C 6B5 Canada; Department of Paediatrics, Western University, 100 Collip Circle, London, ON N6G 4X8 Canada; Department of Clinical Neurological Sciences, Western University, 339 Windermere Road, London, ON N6A 5A5 Canada; Department of Medicine, Western University, 1151 Richmond Str. North, London, ON N6A 3K6 Canada; Department of Medicine, McMaster University, 1280 Main Street West, Hamilton, ON L8S 4K1 Canada; The Thrombosis and Atherosclerosis Research Institute, 237 Barton Str. East, Hamilton, ON L8L 2X2 Canada; Thermo Fisher Scientific, 3747 N Meridian Rd, Rockford, IL 61105 USA; London Regional Genomics Centre, Robarts Research Institute, 1151 Richmond Str. North, London, ON N6A 5B7 Canada; Department of Medical Biophysics, Western University, 1151 Richmond Str. North, London, ON N6A 5C1 Canada

## Abstract

**Introduction:**

Sepsis-associated encephalopathy (SAE) is a state of acute brain dysfunction in response to a systemic infection. We propose that systemic inflammation during sepsis causes increased adhesion of leukocytes to the brain microvasculature, resulting in blood-brain barrier dysfunction. Thus, our objectives were to measure inflammatory analytes in plasma of severe sepsis patients to create an experimental cytokine mixture (CM), and to use this CM to investigate the activation and interactions of polymorphonuclear leukocytes (PMN) and human cerebrovascular endothelial cells (hCMEC/D3) *in vitro*.

**Methods:**

The concentrations of 41 inflammatory analytes were quantified in plasma obtained from 20 severe sepsis patients and 20 age- and sex-matched healthy controls employing an antibody microarray. Two CMs were prepared to mimic severe sepsis (SSCM) and control (CCM), and these CMs were then used for PMN and hCMEC/D3 stimulation *in vitro*. PMN adhesion to hCMEC/D3 was assessed under conditions of flow (shear stress 0.7 dyn/cm^2^).

**Results:**

Eight inflammatory analytes elevated in plasma obtained from severe sepsis patients were used to prepare SSCM and CCM. Stimulation of PMN with SSCM led to a marked increase in PMN adhesion to hCMEC/D3, as compared to CCM. PMN adhesion was abolished with neutralizing antibodies to either β2 (CD18), α_L_/β_2_ (CD11α/CD18; LFA-1) or α_M_/β_2_ (CD11β/CD18; Mac-1) integrins. In addition, immune-neutralization of the endothelial (hCMEC/D3) cell adhesion molecule, ICAM-1 (CD54) also suppressed PMN adhesion.

**Conclusions:**

Human SSCM up-regulates PMN pro-adhesive phenotype and promotes PMN adhesion to cerebrovascular endothelial cells through a β2-integrin-ICAM-1-dependent mechanism. PMN adhesion to the brain microvasculature may contribute to SAE.

## Introduction

Sepsis, a systemic inflammatory response to a known or suspected infection, is a leading cause of admission and mortality in intensive care units (ICUs) [1]. Severe sepsis refers to sepsis accompanied by acute organ dysfunction [2]. Up to 70% of patients with severe sepsis experience an acute neurological dysfunction known as sepsis-associated encephalopathy (SAE) [3]. Acute brain dysfunction in severe sepsis patients is associated with increased rates of mortality and long-term cognitive impairment in survivors [4,5].

The mechanisms of SAE are unknown, but possible instigating factors may be the early activation of the innate immune response and microcirculatory dysfunction that are common manifestations of severe sepsis [6,7]. Activated leukocytes and vascular endothelial cells contribute to systemically increased concentrations of cytokines and chemokines in patients with sepsis [8]. Circulating inflammatory mediator(s) activate polymorphonuclear leukocytes (PMN), leading to up-regulation of the pro-adhesive phenotype and recruitment of PMN to the inflamed tissues and/or organs [9,10]. While PMN are critical for the host defense against pathogens, the systemic activation of PMN also correlates with organ dysfunction in septic patients [11]. Animal models of sepsis demonstrate that overwhelming PMN recruitment is associated with dysfunction of the affected organs, including the brain [12-14].

Leukocyte (such as PMN) recruitment to the microvasculature can be broken down to two general steps: rolling and adhesion [9,15]. Interactions between adhesion molecules expressed constitutively or in response to inflammation, by both endothelial cells and leukocytes, are responsible for cellular interactions. PMN rolling is primarily controlled by a family of glycoproteins (selectins) expressed on both PMN (L-selectin) and endothelial cells (such as P- and E-selectins). Subsequently, PMN firm adhesion is mediated primarily through β-2 integrin (CD18; expressed by PMN) and ICAM-1 (CD54; expressed by endothelial cells) interaction. The β2-integrins form heterodimers with either integrin-αL (CD11a) or integrin-αM (CD11b) to form α_L_/β_2_ (CD11a/CD18; LFA-1) or α_M_/β_2_ (CD11b/CD18; Mac-1), respectively. Both LFA-1 and Mac-1 interact with ICAM-1 to promote PMN adhesion to endothelial cells.

Endothelial cells of the blood-brain barrier (BBB) are likely disrupted during SAE, as vasogenic edema is consistently found on head magnetic resonance imaging in sepsis [16]. Lesions in the subcortical white matter, also consistent with vasogenic edema, are particularly prominent in cases of fatal septic shock [17]. Increased signal associated with contrast agent administration during SAE indicates BBB disruption [6]. The mechanism(s) of BBB disruption in sepsis, however, remain poorly understood.

In this study, we hypothesized that the inflammation associated with severe sepsis would result in greater PMN interactions with the brain microvasculature. Thus, we first aimed to measure circulating inflammatory mediators in blood plasma from patients with severe sepsis, and then investigate the effects of these inflammatory analytes on the pro-adhesive phenotype of human PMNs and human cerebrovascular endothelial cells (hCMEC/D3) *in vitro*.

## Materials and methods

The institutional review boards of Hamilton Health Sciences (Hamilton, ON, Canada) and the University of Western Ontario (London, ON, Canada) approved this study. Consent was obtained from the ICU patients or their legal guardians. Consistency of patient blood sampling in the participating centers was monitored by a study investigator (PCL).

### Severe sepsis patients and blood plasma

Blood plasma collection was described previously [18]. Briefly, blood was collected from ICU patients within the first 24 hours of severe sepsis diagnosis according to the American College of Chest Physicians/Society of Critical Care Medicine (ACCP/SCCM) guidelines [2,19]. Venous blood (4.5 mL) was drawn via indwelling catheters (severe sepsis patients) or fresh venipuncture (from age- and sex-matched healthy controls), and immediately transferred into 15 mL polypropylene tubes (VWR, Mississauga, ON, Canada) containing 0.5 mL of 0.105 mol/L buffered trisodium citrate (pH 5.4) (Sigma-Aldrich, St. Louis, MO, USA) with 100 μL of 1 mol/L benzamidine HCl (approximately 20 mmol/L benzamidine), a serine protease inhibitor (Sigma-Aldrich, St. Louis, MO, USA). The blood was immediately centrifuged at 1,500 g for 10 minutes at 20°C, and the plasma was stored in aliquots at −80°C. Care was taken to ensure that freeze/thaw cycles were avoided. Baseline characteristics of severe sepsis patients were recorded at ICU admission (Table 1). Baseline characteristics included demographics, clinical predictive scores (Acute Physiology and Chronic Health Evaluation II (APACHE II) and multiple organ dysfunction syndrome (MODS)), site of infection, positive blood cultures, and co-morbidities.

**Table 1:** **Baseline characteristics of 20 patients with severe sepsis on ICU day 1**

Age, years	59 ± 4 (20-80)
Gender, female	6 (30%)
APACHE II score	25.8 ± 2.1 (6-42)
MOD score	10.4 ± 0.86 (3-15)
Absolute neutrophil count (ANC)^a^	11.5 ± 0.9 (3.7-19.5)
***Co-morbidities, number (% of total)***	
Cardiovascular	9 (45%)
Pulmonary	5 (25%)
Hepatitis/pancreatitis	5 (25%)
Inflammatory	3 (15%)
Neurological	3 (15%)
Renal	3 (15%)
Diabetes	2 (10%)
***Primary site of infection, number (% of total)***	
Lung	11 (55%)
Abdomen	3 (15%)
Blood	1 (5%)
Urinary Tract	0 (0%)
Other	4 (20%)
Unknown	1 (5%)
***Total number of positive cultures (% of total)*** ^***b***^	
Gram-negative bacteria	10 (50%)
Gram-positive bacteria	9 (45%)
Fungus	2 (10%)

Data are presented as mean ± SE (range) or n (%). ^a^Normal ANC = 1.5-8.0. ^b^Includes blood and non-blood cultures, therefore the total is >100%. Four poly-microbial non-blood cultures were not included in the table. APACHE II, Acute Physiology and Chronic Health Evaluation II; MOD, Multiple organ dysfunction.

### Antibody microarray

Sample analysis was performed by an investigator blinded to the experimental cohort (severe sepsis versus healthy control) in one laboratory (DDF) as batched samples [18]. A total of 4 antibody microarrays were used to analyze the concentrations of 41 different inflammatory mediators in 20 plasma samples from both severe sepsis patients and age- and sex-matched healthy controls. Plasma samples were each diluted 1:3 in Array Sample Diluent and were run on a Thermo Scientific ExcelArray™ (Rockford, IL, USA): Inflammation I (product number 82002), Inflammation II (product number: 82003), Chemotaxis (product number: 82006), and Angiogenesis (product number: 82004) [20].

Protein concentrations were determined for the following human analytes: angiogenin, basic fibroblast growth factor (FGFβ), cysteine-cysteine chemokine ligand 1 (CCL1; I-309), eotaxin, epidermal growth factor (EGF), fas ligand (FasL), granulocyte colony-stimulating factor (G-CSF), granulocyte-macrophage colony-stimulating factor (GM-CSF), growth-regulated oncogene alpha (GRO-α), heparin-binding epidermal growth factor (HB-EGF), hepatocyte growth factor (HGF), interferon gamma (IFNγ), interferon-inducible protein 10 kDa (IP-10), interleukin-1 alpha (IL-1α), interleukin-1 beta (IL-1β), interleukin-2 (IL-2), interleukin-3 (IL-3), interleukin-4 (IL-4), interleukin-5 (IL-5), interleukin-6 (IL-6), interleukin-7 (IL-7), interleukin-8 (IL-8), interleukin-10 (IL-10), interleukin-12 (IL-12), interleukin-13 (IL-13), interleukin-15 (IL-15), interleukin-17 (IL-17), keratinocyte growth factor (KGF), macrophage-derived chemokine (MDC), macrophage inflammatory protein-1 alpha (MIP-1α), macrophage inflammatory protein-1 beta (MIP-1β), monocyte chemotactic protein-1 (MCP-1), monocyte chemotactic protein-3 (MCP-3), neutrophil-activating protein-2 (NAP-2), normal T-cell expressed and secreted (TARC), placenta growth factor (P1GF), regulated upon activation, normal T cell expressed and secreted (RANTES), tissue inhibitor of metalloproteinase 1 (TIMP-1), tumor necrosis factor alpha (TNF-α), tumor necrosis factor beta (TNF-β), and vascular endothelial growth factor (VEGF).

Briefly, 100 μL of multiplexed standards (1,000-12.3 pg/mL) or diluted plasma was applied to each well and incubated at room temperature on a shaker at 200 rpm for two hours. After sample incubation, the microarrays were rinsed three times with wash buffer. Prior to re-incubation on a shaker for one hour, 75 μL pre-titered biotinylated detector antibodies were added to each well. Microarrays were washed three times and 100 μL Streptavidin DyLight 649 (part of Thermo Scientific ExcelArray™kit; Thermo Fisher Scientific, Rockford, IL, USA) was applied to each well, preceding a final incubation of 30 minutes on a shaker. Microarrays were washed five times and dipped briefly in a final rinse solution. Slides were then centrifuged at approximately 200 g until dry. Methodologies for microarray production and antibody cross reactivity assays have been described previously [20]. Cross reactivity on the microarray antibody pairs is ≤5%, as reported by the manufacturer. Microarrays were imaged using an Alphascan™ Microarray Imager (Alpha Innotech, San Leandro, CA, USA) and spot densitometry was performed by Thermo Fisher (Rockford, IL, USA) using ArrayVision™ Software (GE Healthcare, Piscataway, NJ, USA).

### Human cytokine mixtures

The most up-regulated (*P* <0.001) human analytes detected in the plasma of severe sepsis patients (IL-8, G-CSF, IP-10, GRO-α, HGF, MIP-1β, and MCP-1; Table 2) were selected to make the severe sepsis cytokine mixture (SSCM) and control cytokine mixture (CCM). The sepsis-relevant cytokine IL-6 [21,22] was also significantly up-regulated in the plasma of severe sepsis patients (*P* = 0.017) and was added to each cytokine mixture. Recombinant proteins were suspended in PBS containing 1% BSA at 100X concentration measured in severe sepsis blood plasma or healthy control blood plasma (Table 2). Each cytokine mixture was stored at −20°C until thawed for experiments. To treat cells, each cytokine mixture was diluted at 100X concentration in either PBS or VascuLife® EnGS-Mv (LifeLine cell technology, Walkersville, MD, USA) cell culture medium to the corresponding analyte concentrations measured in control blood plasma or severe sepsis blood plasma, respectively. All human recombinant analytes were obtained from either Ebioscience (San Diego, CA, USA) or Invitrogen (Carlsbad, CA, USA).

**Table 2:** **Plasma concentration of inflammatory analytes used to make the control and severe sepsis cytokine mixtures**

**Analyte**	**Control**	**Severe sepsis**	**Fold increase**	***P*** **value**
MCP-1	41.8 ± 12.1	1067.8 ± 196.4	25	<0.001
G-CSF	29.3 ± 9.5	938.4 ± 278.5	32	<0.001
IL-8	10.0 ± 0.0	211.9 ± 43.4	21	<0.001
HGF	290.3 ± 60.8	3346.8 ± 570.5	12	<0.001
MIP-1β	49.7 ± 12.1	381.1 ± 116.0	8	<0.001
IP-10	214.4 ± 26.8	1552.0 ± 254.5	7	<0.001
GROα	92.5 ± 24.2	637.5 ± 136.3	7	<0.001
IL-6	80.8 ± 31.8	387.6 ± 158.4	5	0.017

Plasma was obtained from severe sepsis patients and compared to plasma from age- and sex-matched healthy controls. Analytes were measured with antibody microarray and presented as mean ± SE. Values are in ng/mL (n = 20 per group). MCP-1-monocyte chemotactic protein-1; GCSF-granulocyte colony-stimulating factor; IL-8- interleukin-8; HGF- hepatocyte growth factor; MIP-1β- macrophage inflammatory protein-1 beta; IP-10- interferon-inducible protein 10 kDa; GRO-α- growth-regulated oncogene alpha; IL-6- interleukin-6.

### Human cerebral microvascular endothelial cells

hCMECs (hCMEC/D3 cell line) were isolated and immortalized by Dr Pierre-Olivier Couraud and his colleagues (INSERM, Paris, France; [23]). The immortalization of CMEC was performed via sequential lentiviral transduction of hTERT and SV40 large T antigen transduction into a primary culture of adult CMEC. The hCMEC/D3 cell line offers a unique opportunity to study human cerebrovascular cells in isolation from other cells, and represents a stable, fully characterized, and well-differentiated line of CMEC.

hCMEC/D3 were grown at 37°C and 5% CO_2_ in VascuLife EnGS-Mv (LifeLine Cell Technology, Walkersville, MD, USA) cell culture medium supplemented with recombinant human EGF (0.2%), EnGS (5 ng/mL), heparin (0.75 U/mL), fetal bovine serum ((FBS) 5%), ascorbic acid (50 μg/mL), L-glutamine (10 mM), and hydrocortisone (1.0 μg/mL) (LifeLine Cell Technology, Walkersville, MD, USA) [24]. Penicillin (100 IU/mL) and streptomycin (100 μg/mL) (Wisent Inc, St Bruno, QC, Canada) were also added to cell culture media. hCMEC/D3 at passage 35-50 were used in all experiments.

### Human polymorphonuclear leukocyte isolation

Human PMN were isolated from venous blood of healthy adults immediately before experiments as previously described by us [24]. Briefly, heparinized whole blood (10 mL; 10 U/mL; Pharmaceutical Partners of Canada, Richmond Hill, ON, Canada) was gently mixed by inversion (10 times) with 5 mL of 3% dextran in PBS (pH 7.4). Sedimentation of red blood cells was performed via incubation of the mixture for 20 minutes at 23°C. Blood plasma was removed and gently added onto 5 mL of Histopaque® lymphocyte separation medium (density of 1.077 g/mL; Sigma-Aldrich, St Louis, MO, USA). Samples were centrifuged for 30 minutes at 400 × g at 23°C. Following centrifugation, the pellet (PMN and remaining red blood cells) was re-suspended in 15 mL cold red blood cell lysis buffer, containing NH4Cl (8.3 g/L), KHCO3 (1.0 g/L), and EDTA (0.14 mM) (all purchased from Sigma-Aldrich, St. Louis, MO, USA) in water, adjusted to a pH of 7.2. After five minutes incubation at 4°C, mixtures were centrifuged at 300 × g for five minutes. PMN were re-suspended in 1 mL PBS (4°C), stored on ice until use, and counted using a hemocytometer (VWR, Mississauga, ON, Canada). This procedure yields a PMN population that is 95 to 98% viable (as measured by trypan blue exclusion) and 98% pure (as measured by acetic acid-crystal violet staining) (both reagents were purchased from Sigma-Aldrich, St. Louis, MO, USA).

### Polymorphonuclear leukocyte adhesion to hCMEC/D3

hCMEC/D3 were grown to confluence in μ-Slide VI^0.4^ parallel flow channels (ibidi; MOFA Global, Ingersoll, ON, Canada) and interacted with PMN as previously described by us [24,25]. Briefly, hCMEC/D3 were seeded at a density of 11,000 cells per channel and grown for 5 days, followed by 5 hours of stimulation with either CCM or SSCM diluted in serum free-VascuLife EnGS-Mv cell culture medium. The CCM and SSCM treatments did not affect hCMEC/D3 viability as assessed by ViaCount Flex (Cedarlane, Burlington, ON, Canada) assay and flow-cytometry approach (data not shown). Each channel was then perfused (that is, washed) in the presence of laminar shear stress of 0.7 dyn/cm^2^ for one minute with VascuLife EnGS-Mv cell culture medium, using a Harvard Apparatus 22-syringe pump (Instech, Plymouth Meeting, PA, USA). Subsequently and continuously, hCMEC/D3 were perfused with freshly isolated PMN (1 × 10^6^ PMN/mL; suspended in serum free-VascuLife EnGS-Mv cell culture medium) that have been stimulated either with CCM or SSCM for 10 minutes. The experiment was visualized and video recorded using a Nikon DIAPHOT-300 inverted microscope (Nikon, Chiyoda-ku, Tokyo, Japan) equipped with a temperature-controlled chamber (37°C) and Panasonic WJ-810 video-camera (Panasonic, Kadoma, Osaka, Japan) connected to an SV-video recorder (Panasonic). For each experiment, 5 random fields of view (0.1 mm^2^ each) were recorded for 30 seconds, starting at 4 minutes after initiation of the PMN perfusion. The recorded PMN-hCMEC/D3 adhesive interactions were video-analyzed to count the number of firmly adherent PMN (stationary for at least five seconds). The average number of adherent PMN was expressed as PMN/0.1 mm^2^.

### Adhesion molecule immuno-neutralization

PMN (2 × 10^7^/mL) suspended in PBS were stimulated with CCM or SSCM at 23°C for 10 minutes in a total volume of 0.2 mL. Subsequently, function neutralizing monoclonal antibodies (all IgG1κ subclass; BioLegend, San Diego, CA, USA) directed against either human β_2_-integrin (clone TS 1/18; 25 μg/mL), human LFA-1 (clone HI111; 5 μg/mL), or Mac-1 (clone ICRF44; 5 μg/mL), were added to the PMN suspension for an additional 15 minutes. An isotype control (IgG1κ; BioLegend, San Diego, CA, USA) antibody was used in parallel experiments at the corresponding concentrations. Following treatment, PMN were immediately added to VascuLife EnGS-Mv cell culture medium to a final concentration of 1 × 10^6^/mL and perfused over hCMEC/D3.

In some experiments, hCMEC/D3 grown in laminar flow micro channels (ibidi GmbH) were stimulated with either CCM or SSCM and subsequently treated with function neutralizing anti-human ICAM-1 (CD54; clone HCD54; 5 μg/mL) or isotype control (both IgG1κ subclass; BioLegend, San Diego, CA, USA) antibodies in VascuLife EnGS-Mv cell culture medium for 30 minutes at 37°C, preceding PMN perfusion.

### RNA isolation, cDNA synthesis and quantitative PCR

hCMEC/D3 were seeded (500,000 per well) on six-well plates and grown for two days, preceding four hours of stimulation with either CCM or SSCM. Following stimulation, hCMEC/D3 were washed three times with PBS and RNA was extracted with 1 mL TRIzol® reagent (Life Technologies, Carlsbad, CA, USA), as per the manufacturer’s protocol except the RNA pellet was washed three times with 75% ethanol. RNA was re-suspended in 20 μL of 10 mM Tris, pH 8. The purity and concentration of RNA was determined using a Biophotometer (Eppendorf, Hamburg, Germany). One microgram of RNA per sample was reverse transcribed using iScript™ Reverse Transcription Supermix (Bio-Rad, Hercules CA, USA) according to the manufacturer’s protocol.

Quantitative PCR (qPCR) was performed using Sso Fast™ Probes Supermix (Bio-Rad, Hercules, CA, USA), according to the manufacturer’s protocol, using cDNA at concentrations previously determined to be within 90 to 110% efficiency. Primers were predesigned (Applied Biosystems, Foster City, CA, USA): *18S* (Hs99999901_s1), glyceraldehyde-3-phosphate dehydrogenase (*GAPDH*; Hs99999905_m1), *ICAM-1* (Hs00164932_m1), and *VCAM-1* (Hs01003372_m1). Real-time PCR was run using a C1000™ Touch Thermal Cycler with CFX96™ Real-Time System (Bio-Rad, Hercules CA, USA). Cycling conditions included one-time enzyme activation at 95°C for 30 seconds, followed by 50 cycles of denaturation at 95°C for 5 seconds, and annealing and extension at 60°C for 15 seconds. Target gene expression was normalized to *GAPDH* and *18S* and was expressed relative to gene expression by hCMEC/D3 treated with serum-free VascuLife EnGS-Mv cell culture media.

### Statistical analysis

Data were analyzed using SigmaStat 3.5 (Systat Software Inc., San Jose, CA, USA). Continuous variables are reported with mean ± SE. Groups were pre-screened for normality and compared with either a Student’s t test or Mann-Whitney U test. Multiple groups were analyzed using one-way analysis of variance (ANOVA) with Tukey’s *post-hoc* test. A *P* <0.05 was considered statistically significant.

## Results

### Patient characteristics and plasma inflammatory biomarkers

The baseline characteristics at ICU admission of the 20 severe sepsis patients from whom blood plasma was collected are presented in Table 1. Of these 20 patients with severe sepsis, 18 had septic shock (90%) and 4 died (20%). Antibody microarray analysis of plasma from severe sepsis and control patients indicate that 13 out of the 41 analytes measured were significantly up-regulated in severe sepsis. The measured concentrations of eight analytes were used to create the cytokine mixtures, both SSCM and CCM (IL-8, G-CSF, IP-10, GRO-α, HGF, MIP-1β, MCP-1, IL-6; Table 2; n = 20 per group). In addition, FGFβ (802.3 ± 402.9 versus 1,573.4 ± 456.9 ng/mL; *P* <0.012), IL-15 (38.6 ± 23.1 versus 256.1 ± 128.7 ng/mL; *P* <0.031), MCP-3 (22.4 ± 4.6 versus 93.9 ± 33.0 ng/mL; *P* <0.011), HB-EGF (21.2 ± 7.8 versus 642.0 ± 254.4 ng/mL; *P* <0.044), and KGF (33.7 ± 9.8 versus 177.1 ± 103.0 ng/mL; *P* <0.046) were also significantly up-regulated in the plasma of severe sepsis patients, but not included in the cytokine mixtures.

### Polymorphonuclear leukocyte adhesion to hCMEC/D3

**Figure 1:**
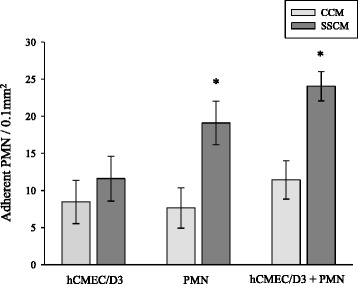
**PMN adhesion to hCMEC/D3 following stimulation with severe sepsis cytokine mixture (SSCM).** hCMEC/D3 were grown on laminar flow microchannels and interacted with PMN following stimulation of hCMEC/D3, PMN, or both PMN and hCMEC/D3, with CCM or SSCM. In these experiments, hCMEC/D3 were stimulated with CCM or SSCM for five hours. PMN were stimulated for 10 minutes immediately before interacting them with hCMEC/D3. PMN adhesion to hCMEC/D3 was assessed in the presence of flow (laminar shear stress 0.7 dyn/cm^2^). n = 7; *P <0.05 as compared to corresponding control cytokine mixture (CCM). CCM-control cytokine mixture; SSCM-severe sepsis cytokine mixture; hCMEC/D3-human cerebral microvascular endothelial cells/D3; PMN-polymorphonuclear leukocytes.

PMN and/or hCMEC/D3 were stimulated with either SSCM or CCM and assessed for PMN adhesion to hCMEC/D3 in the presence of flow (laminar shear stress 0.7 dyn/cm^2^). Stimulation of hCMEC/D3 with SSCM did not significantly increase the adhesion of naïve (unstimulated) PMN (Figure 1). On the contrary, stimulation of PMN with SSCM significantly increased PMN adhesion to hCMEC/D3 (*P* <0.05; n = 7; Figure 1), while simultaneous treatment of PMN and hCMEC/D3 with SSCM (co-stimulation) had no additive effects.

### Role of β2-integrins and ICAM-1 in polymorphonuclear leukocyte adhesion to hCMEC/D3

**Figure 2:**
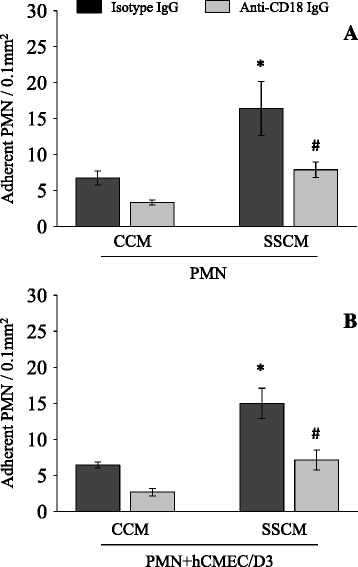
**Effects of anti-β**_**2**_ **-integrin (CD18) antibody on severe sepsis cytokine mixture (SSCM)-induced PMN adhesion to hCMEC/D3.** PMN were first stimulated with CCM or SSCM for 10 minutes and subsequently treated with anti-β_2_-integrin (CD18) function neutralizing antibody (or control isotype antibody) for an additional 15 minutes. PMN adhesion to stimulated PMN **(A)** or stimulated PMN and hCMEC/D3 **(B)** was assessed in the presence of flow (laminar shear stress 0.7 dyn/cm^2^). n = 4. *P <0.05 as compared to corresponding control cytokine mixture (CCM); # *P* <0.05 as compared to isotype IgG treatment. CCM-control cytokine mixture; SSCM-severe sepsis cytokine mixture; hCMEC/D3-human cerebral microvascular endothelial cells/D3; PMN-polymorphonuclear leukocytes; IgG-immunoglobulin G; CD18-cluster of differentiation 18.

**Figure 3:**
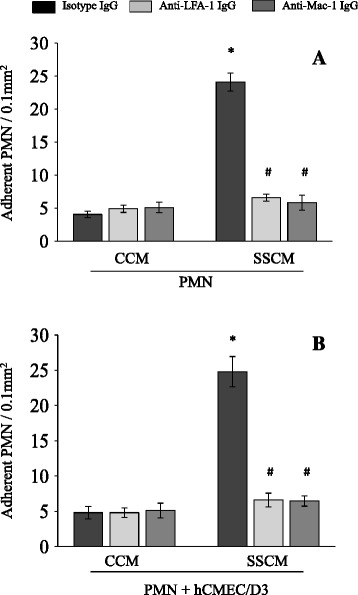
**Effects of anti-LFA-1 and anti-Mac-1 antibodies on severe sepsis cytokine mixture (SSCM)-induced PMN adhesion to hCMEC/D3.** PMN were first stimulated with CCM or SSCM for 10 minutes and subsequently treated with anti-LFA-1 or anti-Mac-1 function neutralizing antibody (or control isotype antibody) for an additional 15 minutes. PMN adhesion to stimulated PMN **(A)** or stimulated PMN and hCMEC/D3 **(B)** was assessed in the presence of flow (laminar shear stress 0.7 dyn/cm^2^). n = 4. *P <0.05 as compared to corresponding control cytokine mixture (CCM); # *P* <0.05 as compared to isotype IgG treatment. CCM-control cytokine mixture; SSCM-severe sepsis cytokine mixture; hCMEC/D3-human cerebral microvascular endothelial cells/D3; PMN-polymorphonuclear leukocytes; IgG-immunoglobulin G; LFA-1 - lymphocyte function-associated antigen 1; Mac-1 - macrophage-1 antigen.

Antibody neutralization was used to assess the role of β2-integrins on SSCM-induced PMN adhesion to hCMEC/D3. SSCM-stimulated PMN were treated with either a function neutralizing anti-β_2_-integrin antibody or isotype control antibody before hCMEC/D3 interaction. Anti-β_2_-integrin antibody, but not isotype matching control antibody, prevented PMN adhesion to naïve (unstimulated) hCMEC/D3 (*P* <0.05; n = 4; Figure 2A). The anti-β_2_-integrin antibody was also effective in preventing SSCM-induced PMN adhesion to SSCM-stimulated hCMEC/D3 (*P* <0.05; n = 4; Figure 2B). Finally, neutralizing antibodies revealed that both α_L_/β_2_ (CD11a/CD18; LFA-1) and α_M_/β_2_ (CD11b/CD18; Mac-1) integrins equally contribute to SSCM-induced PMN adhesion to hCMEC/D3 (*P* <0.05; n = 4; Figure 3).

**Figure 4:**
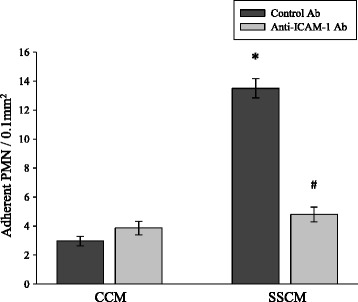
**Effects of anti-ICAM-1 antibody on severe sepsis cytokine mixture (SSCM)-induced PMN adhesion to hCMEC/D3.** hCMEC/D3 were stimulated with CCM or SSCM for 5 hours, treated with anti-ICAM-1 function neutralizing antibody for 30 minutes, and interacted with CCM- or SSCM-stimulated (10 minutes) PMN to assess PMN adhesion in the presence of flow (laminar shear stress 0.7 dyn/cm^2^). n = 6; *P <0.05 as compared to corresponding control cytokine mixture (CCM); # *P* <0.05 as compared to isotype IgG treatment. CCM-control cytokine mixture; SSCM-severe sepsis cytokine mixture; hCMEC/D3-human cerebral microvascular endothelial cells/D3; PMN-polymorphonuclear leukocytes; Ab-antibody; ICAM-1 – intercellular adhesion molecule-1.

**Figure 5:**
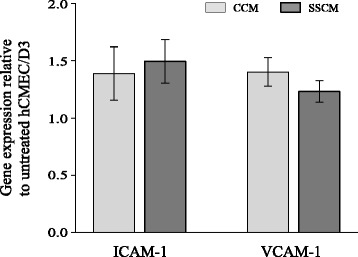
**Effects of severe sepsis cytokine mixture (SSCM) on adhesion molecule ICAM-1 and VCAM-1 expression in hCMEC/D3.** hCMEC/D3 were stimulated for 4 hours with either CCM or SSCM preceding analysis of *VCAM-1* and *ICAM-1* gene expression using qPCR. Data is presented as gene expression normalized to *GAPDH* and *18S* expression, relative to hCMEC/D3 treated with serum-free VascuLife EnGS-Mv cell culture media (n = 5). CCM-control cytokine mixture; SSCM-severe sepsis cytokine mixture; hCMEC/D3-human cerebral microvascular endothelial cells/D3; ICAM-1 – intercellular adhesion molecule-1; VCAM-1 – vascular cell adhesion molecule-1; 18S-18S ribosomal RNA; GAPDH-glyceraldehyde 3-phosphate dehydrogenase; qPCR- quantitative polymerase chain reaction.

Interfering with hCMEC/D3 ICAM-1 function by pre-treatment of hCMEC/D3 with an anti-ICAM-1 neutralizing antibody was also effective in reducing adhesion of SSCM-stimulated PMN to SSCM-stimulated hCMEC/D3 (*P* <0.05; n = 6; Figure 4). The reduced PMN adhesion to hCMEC/D3 following immune-neutralization of ICAM-1 was likely due to interference with the constitutively expressed levels of ICAM-1 on the surface of hCMEC/D3, as SSCM failed to up-regulate ICAM-1 expression as assessed by qPCR (Figure 5).

## Discussion

The present study identified an inflammatory profile in severe sepsis patients at ICU day one that up-regulated a pro-adhesive phenotype of PMN, resulting in increased adhesion to hCMEC/D3 *in vitro*. These effects may be important for the pathophysiology of SAE, as adhered and/or activated PMN could result in dysfunction of the BBB.

The extracellular environment of the brain is highly regulated by the BBB. Dysfunction of the BBB has been demonstrated with brain imaging in patients with SAE [6,16,17], and BBB dysfunction is also observed in lipopolysaccharide-challenged animals [26]. Injury to the cellular components of the BBB, including cerebrovascular endothelial cells [27], pericytes [28], and astrocytes [29], was observed in experimental animal models of sepsis. Cerebrovascular endothelial cell dysfunction may lead to dysregulation of the brain extracellular environment and subsequent neuronal dysfunction, thereby causing SAE.

In the current study, we collected blood plasma samples from ICU patients within the first 24 hours of severe sepsis diagnosis. Encephalopathy occurs early in sepsis, often developing on the first day of ICU admission [30]. Thus, blood was sampled at a critical time point for SAE development.

A total of 13 inflammatory analytes were up-regulated in blood plasma obtained from severe sepsis patients compared to blood plasma from age- and sex-matched healthy controls. Most of the up-regulated analytes are pro-inflammatory cytokines (such as IL-6) and CXC- and CC-family chemokines (such as IL-8, GRO-α, MCP-1, and MIP-1β), with well-documented roles in cellular activation and leukocyte recruitment to vascular endothelium [31,32]. In addition, a marked increase in cell growth-modulating factors (such as HGF, FGFβ, HB-EGF, and KGF) in the circulation of severe sepsis patients was also found. The role of these growth factors in tissue remodelling has been shown previously, but the exact role of these molecules in the pathophysiology of severe sepsis is unknown.

Acute expression of individual cytokines and chemokines (such as IL-6, IL-8, MCP-1, and G-CSF) has been reported to correlate with the presence and severity of organ dysfunction in patients with severe sepsis and septic shock [22]. Interestingly, cytokines that are most commonly employed in experimental models of inflammation and/or sepsis (TNF-α, IL-1β, and IFN-γ) were not significantly increased in severe sepsis plasma obtained within 24 hours of ICU admission. One possible explanation for the latter phenomenon could be that the cytokines mentioned above may be expressed more prominently at different stages (for example, earlier or later than 24 hours post-admission time) and severities of sepsis, or in subgroups of severe sepsis patients [22,33]. Thus, it would be plausible to assume that the severe sepsis analyte profile within the first 24 hours of ICU admission represents not an ‘early’, but rather a ‘later’ cytokine response, that may intensify and perpetuate the inflammatory response.

One unique aspect of this study was the use of SSCM that consisted of eight cytokines and chemokines at the actual concentrations detected in the plasma of severe sepsis patients. In contrast, previous sepsis studies in the field have used a single cytokine and chemokine-based stimulation of the cells at often ‘un-physiologically’ high concentrations. In the current study we employed a severe sepsis analyte profile obtained at a time-point of disease that is clinically relevant to SAE development [3]. Our results indicate that PMN, but not hCMEC/D3, are more responsive to SSCM, with regards to up-regulation of the pro-adhesive phenotype. Stimulation of PMN, or PMN and hCMEC/D3, with SSCM resulted in an increased adhesion of PMN to hCMEC/D3 under *in vitro* conditions of flow, whereas stimulation of hCMEC/D3 only had no effect on PMN adhesion. This latter phenomenon may be explained by the inability of SSCM to up-regulate inducible adhesion molecule (such as ICAM-1 or VCAM-1) expression in hCMEC/D3 (as evidenced by qPCR analysis; Figure 5). On the other hand, the levels of constitutively expressed ICAM-1 on the surface of endothelial cells are sufficient to promote adhesion of activated PMN. Whether cerebrovascular endothelial cells (such as hCMEC/D3) are less sensitive to the pro-inflammatory cytokine and chemokine stimulation at physiologic concentrations as compared to the non-brain endothelial cells (such as pulmonary microvascular endothelial cells or umbilical vein endothelial cells) remains to be determined.

Our data indicate that β_2_-integrins (such as LFA-1 and Mac-1) are required for SSCM-induced PMN adhesion to hCMEC/D3. Interestingly, immune-neutralization of ICAM-1 (a receptor for β_2_-integrins) in hCMEC/D3 was also very effective in reducing PMN adhesion, despite the fact that SSCM failed to up-regulate ICAM-1 expression in hCMEC/D3. The latter may be explained by reduced availability of the constitutively expressed ICAM-1 following treatment of hCMEC/D3 with the immunoneutralizing anti-ICAM-1 antibody.

Up-regulated surface expression of β_2_-integrin has been reported on freshly isolated PMN stimulated with blood plasma obtained from severe sepsis patients 24 hours after diagnosis [34]. Treatment of PMN with individual chemokines, such as IL-8, may also induce LFA-1- or Mac-1-dependent PMN adhesion [35,36]. The up-regulation of β_2_-integrins (such as LFA-1 and Mac-1) surface expression is not always a prerequisite for LFA-1- and Mac-1-dependent adhesion. Rather, β_2_-integrins that are constitutively expressed on the leukocyte cell membrane undergo conformational activation to a pro-adherent state that promotes leukocyte adhesion to molecules such as ICAM-1 [37,38]. LFA-1 and Mac-1 are known to bind ICAM-1 on vascular endothelial cells to facilitate PMN adhesion [39,40]. Ameliorated PMN recruitment to brain tissue has been demonstrated during endotoxemia in ICAM-1-deficient mice, similar to our findings in which an anti-ICAM-1 antibody reduced PMN adhesion to hCMEC/D3, suggesting an important role for β_2_-integrin-ICAM-1 interactions in PMN adhesion to CMEC [41].

There is currently no data published regarding the use of antibody treatment directed against LFA-1, Mac-1, or β_2_-integrin in humans for treatment of severe sepsis or SAE. However, LFA-1- and Mac-1-dependent recruitment of PMN is not unique to the brain, and studies that employed murine and rabbit models of sepsis have demonstrated that antibodies directed against β_2_-integrin, LFA-1, and Mac-1 can reduce PMN recruitment and ameliorate subsequent dysfunction of lung and liver [42-44].

Whether PMN adhesion to the brain microvasculature in severe sepsis causes brain dysfunction is unclear. However, PMN are armed with many enzymes involved in host defense against pathogens, including NADPH oxidase, myeloperoxidase (MPO), matrix metalloproteinases (MMPs), elastase, and cathepsins [45]. Overwhelming local recruitment of activated PMN to brain microvasculature could expose CMEC to the deleterious effects of these enzymes. Both NADPH oxidase and MPO produce reactive oxygen species (ROS), which directly or indirectly contribute to the disruption of vascular endothelial cell intercellular junctions [27,46,47]. ROS production by PMN isolated from severe sepsis patients, and elevated MPO concentrations in blood plasma isolated from septic patients have both been reported [11,48]. MMPs and elastase can also damage endothelial cell adherens and tight junctions [49,50]. Disruption of the brain microvasculature may contribute to pathological activation of glial cells or neurons and, subsequently, to the development of SAE [27,28].

At present, there is little pathophysiological data on SAE, and no studies using human tissues. Animal models of sepsis are a predominant source of data regarding potential mechanisms of brain dysfunction in sepsis. Excessive PMN recruitment, associated with neurological impairment in the brains of septic mice, has been reported [13,51]. PMN recruitment was ameliorated by treatment with antibodies directed against ICAM-1, β_2_-integrin, and P-selectin adhesion molecules. Anti-P-selectin antibody treatment was associated with decreased sickness behaviour by septic mice, suggesting a possible role for PMN-endothelial interactions in brain dysfunction during sepsis.

Our data have several limitations. First, our *in vitro* culture model may not fully represent the cerebral microvasculature in severe sepsis. Brain endothelial cells *in vivo* are in close contact with astrocytes, and co-culture of hCMEC/D3 with astrocytes has been demonstrated to decrease basal protein expression of ICAM-1 [52]. Therefore, hCMEC/D3 may express ICAM-1 at a higher basal rate compared to brain endothelial cells *in vivo*, which may reduce the window for up-regulation of ICAM-1 in response to cytokine stimulation. Second, our SSCM does not include all potential mediators that may play a role in leukocyte and/or endothelial activation in inflammation. The use of SSCM, however, provides valuable information regarding the acute inflammatory state in severe sepsis patients, at a time point when brain dysfunction most often occurs [3,53].

## Conclusions

Our data elucidate the late inflammatory changes in plasma during the first 24 hours of severe sepsis diagnosis in ICU patients, and demonstrate inflammation-induced PMN adhesion to hCMEC/D3, mediated by β_2_-integrin-ICAM-1 interaction.

## Key messages

- The pathophysiology of sepsis-associated encephalopathy (SAE) is unknown, but systemic inflammation may cause dysfunction of the brain microvasculature.
- We measured inflammatory analytes in plasma during severe sepsis, and created a severe sepsis cytokine mixture (SSCM).
- SSCM induced neutrophilic leukocyte (PMN) adhesion to cultured cerebrovascular endothelial cells *in vitro*.
- β2-integrins on PMNs, and ICAM-1 on cerebrovascular endothelial cells, mediated increased cellular adhesions.
- PMN adhesion to the brain microvasculature during severe sepsis may contribute to SAE.

## Abbreviations

ACCP/SCCM: American College of Chest Physicians/Society of Critical Care Medicine
ANOVA: Analysis of variance
APACHE II: Acute Physiology and Chronic Health Evaluation II
BSA: bovine serum albumin
CCM: control cytokine mixture
CD: cluster of differentiation
CMEC: cerebral microvascular endothelial cells
EDTA: ethylenediaminetetraacetic acid
EGF: epidermal growth factor
EnGS-Mv: endothelial growth supplement-microvascular
FBS: fetal bovine serum
G-CSF: granulocyte colony-stimulating factor
GRO-α: Growth-regulated oncogene α
hCMEC/D3: human cerebral microvascular endothelial cells
hTERT: human telomerase reverse transcriptase
HGF: hepatocyte growth factor
ICAM-1: intercellular adhesion molecule 1
ICU: intensive care unit
IgG: immunoglobulin G
IL: interleukin
IP-10: interferon-inducible protein 10 kDa
LFA-1: lymphocyte function-associated antigen 1
Mac-1: macrophage-1 antigen
MCP-1: monocyte chemotactic protein-1
MIP-1β: macrophage inflammatory protein-1 beta
MMP: matrix metalloproteinase
MODS: multiorgan dysfunction syndrome
MPO: myeloperoxidase
NADPH: Nicotinamide adenine dinucleotide phosphate
PBS: phosphate buffered saline
PMN: polymorphonuclear leukocytes
qPCR: quantitative real-time polymerase chain reaction
ROS: reactive oxygen species
SAE: sepsis-associated encephalopathy
SSCM: severe sepsis cytokine mixture
SV40: Simian vacuolating virus 40
